# The chemical components, action mechanisms, and clinical evidences of YiQiFuMai injection in the treatment of heart failure

**DOI:** 10.3389/fphar.2022.1040235

**Published:** 2022-11-24

**Authors:** Shichao Lv, Yunjiao Wang, Wanqin Zhang, Hongcai Shang

**Affiliations:** ^1^ Key Laboratory of Chinese Internal Medicine of MOE, Dongzhimen Hospital, Beijing, China; ^2^ Department of Geriatrics, First Teaching Hospital of Tianjin University of Traditional Chinese Medicine, Tianjin, China

**Keywords:** heart failure, YiQiFuMai injection, chemical components, clinical evidences, mechanisms

## Abstract

YiQiFuMai injection (YQFM), derived from Shengmai Powder, is wildly applied in the treatment of cardiovascular diseases, such as coronary heart disease and chronic cardiac insufficiency. YiQiFuMai injection is mainly composed of Radix of *Panax ginseng* C.A. Mey. (Araliaceae), Radix of *Ophiopogon japonicus* (Thunb.) Ker Gawl (Liliaceae), and Fructus of *Schisandra chinensis* (Turcz.) Baill (Schisandraceae), and Triterpene saponins, steroidal saponins, lignans, and flavonoids play the vital role in the potency and efficacy. Long-term clinical practice has confirmed the positive effect of YiQiFuMai injection in the treatment of heart failure, and few adverse events have been reported. In addition, the protective effect of YiQiFuMai injection is related to the regulation of mitochondrial function, anti-apoptosis, amelioration of oxidant stress, inhibiting the expression of inflammatory mediators, regulating the expression of miRNAs, maintaining the balance of matrix metalloproteinases/tissue inhibitor of metalloproteinases (MMP/TIMP) and anti-hypoxia.

## 1 Introduction

Heart failure whose incidence rate rises year by year, is the late stage of cardiovascular disease with high mortality and rehospitalization rates. The latest epidemiological survey showed that the prevalence of heart failure in China was 1.3% (an estimated 13.7 million patients), which had increased by 44% in the past 15 years (Hao et al., 2019). And the mortality rates of heart failure within 30 days, 1 year and 5 years after hospitalization were 10.4%, 22% and 42.3% respectively. The rehospitalization rates within 1 year among patients with acute and chronic heart failure were 43.9% and 31.9% respectively (Loehr et al., 2008; Maggioni et al., 2013). As the last battlefield for the prevention and treatment of cardiovascular diseases, heart failure has caused the heavy economic burden to society and joined the rank of grievous public health problems. So far, pharmacotherapy aiming at blocking the renin angiotensin aldosterone system and sympathetic nervous system is the cornerstone in the treatment of heart failure. And new drugs have earned a place, such as angiotensin receptor neprilysin inhibitor (ARNI) and sodium glucose cotransporter two inhibitor (SGLT2i) (McDonagh et al., 2021). Although great progress has realized in the prevention and treatment of heart failure, the overall prognosis is still poor, and the 5-year survival rate is equivalent to that of some malignant tumors (Stewart et al., 2001). Exploring effective and thorough strategy for the treatment of heart failure remains to be done.

Traditional Chinese Medicine, based on the concept of holism, differentiates syndromes and gives treatment in a Multi-target and individualized way, and its advantages lie in increasing exercise tolerance, improving the quality of life, elevating cardiac function, delaying myocardial remodeling, reducing mortality and rehospitalization rates (Zhang and Li, 2014; Sun et al., 2016). Shengmai Powder, first described in a classic named ‘*Yi Xue Qi Yuan*’, is composed of Radix of *Panax ginseng* C.A. Mey. (Araliaceae), Radix of *Ophiopogon japonicus* (Thunb.) Ker Gawl (Liliaceae), and Fructus of *Schisandra chinensis* (Turcz.) Baill (Schisandraceae), which has effects of replenishing qi, recovering pulse, nourishing yin, and promoting body fluid production (Zhang, 1978). It is commonly applied in the treatment of heart failure, coronary heart disease, hypertension, and viral myocarditis (Wu, 1997). Chinese patent medicines derived from Shengmai Powder include YiQiFuMai injection (YQFM), Shengmai injection, Shenmai injection, Shengmai San, Shengmai Yin, Shengmai capsule, *etc.* Studies have demonstrated the cardioprotective effects of Shengmai-related formulas, inccluding improving cardiac function, ameliorating ventricular remodeling, suppressing inflammation, reducing collagen deposition, and inhibiting apoptosis (Cao Z. H. et al., 2019; Yin et al., 2020).

YQFM, the product of Traditional Chinese Medicine combined with modern pharmaceutical technology, not only retains the effecy of Shengmai Powder, but also takes effect more quicky (Du et al., 2021). It is the only powder injection traditional Chinese medicine cardiotonic approved for listing by the state at present (Fu et al., 2020). YQFM is mainly comprised of Radix of *Panax ginseng* C.A. Mey. (Araliaceae), Radix of *Ophiopogon japonicus* (Thunb.) Ker Gawl (Liliaceae), and Fructus of *Schisandra chinensis* (Turcz.) Baill (Schisandraceae). Modern pharmacological studies have shown that, as the main active substance of Radix of *Panax ginseng* C.A. Mey. (Araliaceae), ginsenosides effectively inhibited myocardial hypertrophy, improved myocardial ischemia, promoted vascular regeneration and inhibited apoptosis (Wang W. et al., 2016). Research has found that Radix of *Ophiopogon japonicus* (Thunb.) Ker Gawl (Liliaceae) guarded cardiovascular system by resisting myocardial ischemia, arrhythmia, thrombosis and improving microcirculation (Fan et al., 2020). The study showed that Fructus of *Schisandra chinensis* (Turcz.) Baill (Schisandraceae) acted on various signal pathways to protect myocardial cells from inflammation, apoptosis, oxidative stress, atherosclerosis and other advert effects (Xing et al., 2021). Therefore, YQFM can improve heart function and alleviate heart failure by reducing myocardial ischemia-reperfusion injury, antioxidant stress, regulating ventricular remodeling, and reducing the release of inflammatory factors (Ju et al., 2018). In clinical practice, it is mainly used for the treatment of heart failure, coronary heart disease, angina pectoris and other cardiovascular diseases (Zhang et al., 2021). In 2007, China Food and Drug Administration approved YQFM for the treatment of cardiovascular diseases, including coronary heart disease, exertional angina pectoris and chronic cardiac insufficiency. And YQFM is recommended for acute exacerbation of heart failure in the ‘expert consensus on diagnosis and treatment of chronic heart failure with integrated traditional Chinese and Western medicine’ (Chinese Association of Integrative Medicine, 2016). Research has showed that YQFM significantly reduced the level of N-terminal pro-B-type natriuretic peptide (NT-proBNP), improved cardiac function, and relieved symptoms and signs in patients with acute heart failure (Wang et al., 2021). This review summarized the components, mechanisms and clinical evidences of YQFM in the treatment of heart failure in order to provide a theoretical basis for clinical practice.

### 1.1 Chemical components of YiQiFuMai injection in the treatment of heart failure

YQFM is mainly comprised of Radix of *Panax ginseng* C.A. Mey. (Araliaceae), Radix of *Ophiopogon japonicus* (Thunb.) Ker Gawl (Liliaceae), and Fructus of *Schisandra chinensis* (Turcz.) Baill (Schisandraceae). Triterpene saponins are the main components of Radix of *Panax ginseng* C.A. Mey. (Araliaceae). In addition, steroidal saponins, flavonoids, and carbohydrates are the main components of Radix of *Ophiopogon japonicus* (Thunb.) Ker Gawl (Liliaceae) while lignans compounds are the main components of Fructus of *Schisandra chinensis* (Turcz.) Baill (Schisandraceae) (Table 1). With the development of analytical methods such as high-performance liquid chromatography (HPLC), liquid-mass spectrometry (LMS) and metabolomics, these new technologies have been applied to explore the chemical components of YQFM. For instance, Zhou et al. have identified the components of QYFM with the help of liquid chromatography electrospray ionization mass spectrometry (LC-ESI-MS), including ginsenoside Rf, Rb1, Rb2, Rb3, Rd, Rg3, 20 (S), ginsenoside F2, and Schisandrin B (Zhou et al., 2009). Liu et al. have identified 21 saponins in YQFM by HPLC coupled with quadrupole time-of-flight tandem mass spectrometry (HPLC-Q-TOF-MS), and among them, 13 saponins were reported for the first time (Liu et al., 2018). Furthermore, Wang et al. have determined the contents of fructose, glucose, sucrose, and maltose in YQFM by HPLC-evaporative light scattering and electrospray (Wang Y. et al., 2020). Liu et al. not only identified 65 compounds of YQFM by ultrafast liquid chromatography-ion trap time-of-flight MS (UFLC-IT-TOF/MS) but also quantitatively analyzed 21 compounds, including three Ophiopogon japonicus, 15 ginsenosides, and three lignans (Liu et al., 2016). Zhou et al. have determined 145 compounds of YQFM by ultra-performance liquid chromatography coupled with quadrupole time-of-flight tandem mass spectrometry (UPLC-Q-TOF/MS), including 20 flavonoids, 24 lignans, 27 saponins, 15 carbohydrates, 38 organic acids, and 22 sterols, peptides, and esters (Zhou et al., 2018). Additionally, using saikosaponin A as the internal reference, Zhou et al. detected nine ginsenosides in the plasma of Wistar rats after the intravenous injection of YQFM by LC-electrospray ionization (ESI)-MS (Zhou et al., 2011). Zhang et al. reported eight ginsenosides and four lignans detected by LC-ESI-MS/MS in the plasma of Beagle dogs after intravenous injection of YQFM (Zhang et al., 2018). According to the research of Zheng et al., 10 ginsenosides were identified by UFLC-MS/MS in the plasma of rats with chronic heart failure after the intervention of YQFM (Zheng et al., 2018). From the view of chemical components, pharmacodynamics, network pharmacology, pharmacokinetics, and properties, Li et al. has demonstrated quality markers of YQFM, including ginsenosides (Rb1, Rg1, Rf, Rh1, Rc, Rb2, Ro, and Rg3), Ophiopogon saponin C, phiogenin-3-O-α-l-rhamnopyranosyl-(1→2)-β-d-glucopyranoside, pennogenin-3-O-α-l-rhamnopyranosyl-(1→2)-β-d-xylopyranosyl-(1→4)-β-d-glucopyranoside, fructose, and schisandrin, representing the transfer and change of main substances during the preparation of YQFM, which are the main medicinal chemical components of YQFM (Li D. K. et al., 2019).

**TABLE 1:** Chemical components of YiQiFuMai injection.

Types	Components	Ref
Triterpenoid saponins	Ginsenoside Rf, Ginsenoside Rb_1_, Ginsenoside Rb_2_, Ginsenoside Rb_3_, Ginsenoside Rg_3_, Ginsenoside Rd, 20(S)-ginsenoside F_2_, 20-glc-Ginsenoside Rf, Notoginsenoside R_1_, Ginsenoside Re, Ginsenoside Ra_3_, Ginsenoside F_3_, Ginsenoside Ra_1_, Ginsenoside Rg_1_, Ginsenoside Rg_2_, Ginsenoside Rh_1_, Ginsenoside Rc, Ginsenoside Rd, Notoginsenoside R_2_, S-Ginsenoside Rg_2_, S-Ginsenoside Rg_3_, R-Ginsenoside Rg_3_, S-Ginsenoside Rs_3_, Ginsenoside F_1_, Ginsenoside F_2_, Ginsenoside Ro, Saponin Rb-2, Chikusetsu saponin Iva, ManoylGinsenoside, S-Ginsenoside Rh_2_, R-Ginsenoside Rh_2_, et al	Zhou et al. (2009) Liu et al. (2018); Liu et al. (2016); Zhou et al. (2018); Zhou et al. (2011); Zhang et al. (2018); Zheng et al. (2018)
Steroidal saponins	Ophiopojaponin C, Ophiopojaponin B, Ophiopojaponin E, Ophiopojaponin Ra, Ophiopojaponin A, 14-hydroxyophiopojaponin C, Ophiogenin-3-O-α-l-rhamnopyranosyl-(1→2)-β-d-glucopyranoside, Ophiogenin-3-O-α-l-rhamnopyranosyl-(1→4)-β-d- xylopyranosyl-(1→4)-β-d-glucopyranoside, Pennogenin-3-O-α-l-rhamnopyranosyl-(1→2)-β-d-xylopyranosyl-(1→4)-β-d-glucopyranoside, et al	Liu et al. (2018); Liu et al. (2016); Zhou et al. (2018)
Lignans	Schizandrol A, Schizandrol B, Gomisin D, Gomisin M_1_, Gomisin N, g-schizandrol, Gomisin E, Gomisin S, Tigloylaomisin H, Isoschizandrol, Schisantherin A, Schizantherin B, Schizantherin C, Benzoylgomisin Q, Gomisin O, Gomisin L_1_, Gomisin F, Gomisin J, Gomisin K_1_, et al	Zhou et al. (2009); Liu et al. (2018); Liu et al. (2016); Zhou et al. (2018); Zhang et al. (2018)
Flavones	Ophiopogonone E, Ophiopogonone B, 6-aldehydo-isophiopogonone, Ophiopogone A, Ophiopogonanone A, Methylophiopogonone A, Methylophiopogonone B, 2′-hydroxyisoophiopogonin A, et al	Liu et al. (2016); Zhou et al. (2018)
Sugars	Fructose, Glucose, Sucrose, Maltose, et al	Wang Y. et al. (2020)

### 1.2 Action mechanisms of YiQiFuMai injection in the treatment of heart failure

Heart failure is a chronic and progressive disease, and myocardial remodeling is a critical factor in the initiation and progression of heart failure.

#### 1.2.1 Improving cardiac function

In HF mice induced by the permanent coronary artery ligation (CAL) with the intervention of YQFM for 2 weeks (0.13 g/kg, 0.26 g/kg, and 0.53 g/kg) showed that YQFM (0.53 g/kg) improved left ventricular function and ameliorated structural injury. It was reported that YQFM restrained the activity of serum lactic dehydrogenase (LDH) and creatine kinase (CK), lowered the levels of serum malondialdehyde (MDA), amino-terminal pro-peptide of pro-collagen type III (PIIINP), NT-proBNP, and myocardial hydroxyproline (HYP). And YQFM appears to reduce oxidation stress, suppress myocardial collagen deposition and fibrosis, and ameliorate cardiac remodeling by the of the blocking effect on mitogen-activated protein kinases (MAPKs) signaling pathway (Pang et al., 2017). Wistar rats were subjected to abdominal aortic coarctation to establish a chronic heart failure model. And the indications for successful modeling was determined by LVEF% ≤ 60% at 8 weeks after the operation. After 14 days of continuous treatment with YQFM (520, 260 and 1,040 mg/kg), the results indicated that YQFM increased the left ventricular posterior wall in the systolic (LVPWs), ejection fraction (EF), fractional shortening (FS), reduced left ventricular end-systolic diameter (LVESD), the levels of serum brain natriuretic peptide (BNP) and copeptin (CPP), whereby improving cardiac function and delaying ventricular remodeling in rats (Zhang et al., 2015).

#### 1.2.2 Reducing myocardial injury

SD rats were given the intervention of YQFM (0.28, 0.55 and 1.10 g/kg) *via* tail vein injection for 7 days followed by the intraperitoneal injection of doxorubicin (25 mg/kg) for 5 days to establish acute myocardial injury model. The results manifested that YQFM alleviated doxorubicin-induced myocardial injury and improved cardiac function in rats by reducing the serum levels of LDH, CK, and AST, decreasing left ventricular end-diastolic diameter (LVEDD), and elevating FS (Wang XD. et al., 2020). Vitro experiment has also confirmed the cardioprotective effects of YQFM that the application of YQFM (5 mg/ml) boosted the viability of H9c2 cells exposed to H_2_O_2_ (0.2 mmol/L, 5 h) (Li et al., 2020).

#### 1.2.3 Improving mitochondrial function

ICR mice were treated with different concentrations of YQFM (0.13, 0.26 and 0.53 g/kg, intraperitoneal) for 2 weeks after CAL. The results showed that YQFM redressed mitochondrial dysfunction by normalizing mitochondrial morphology, increasing mitochondrial membrane potential (Δψm), inhibiting the generation of reactive oxygen species (ROS), up-regulating the expression of mitochondrial fusion protein 2 (Mfn2), and reducing the phosphorylation of dynamin-related protein 1 (Drp1), which was related to reduction in NADPH oxidase 2 (NOX2), p67^phox^, NOX4, calcium voltage-gated channel subunit α1C (CACNA1C) and phosphorylation of calmodulin dependent protein kinase II (p-CaMKII) (Zhang et al., 2019).

#### 1.2.4 Ameliorating myocardial apoptosis

In HF mouse models induced by CAL, after the intraperitoneal injection of YQFM (0.13 g/kg, 0.26 g/kg, 0.53 g/kg) for 14 days, the results showed that the levels of serum creatine kinase-MB (CK-MB), aspartate aminotransferase (AST), interleukin-6 (IL-6), troponin, myosin, and myoglobin were down-regulated, and the omentin level elevated. And the study indicated that YQFM improved left ventricular systolic function and suppressed apoptosis on account of boosting the expression of phosphatidylinositol 3-kinase (PI3K), the phosphorylation of protein kinase B (Akt) and adenine monophosphate activated protein kinase (AMPK) and inhibiting the phosphorylation of p38, C-Jun Kinase enzyme (JNK), and extracellular signal-regulated kinase 1/2 (ERK1/2) (Li F. et al., 2019). *In vitro* experiments, compared with the control group, namely injured H9c2 cells induced by doxorubicin (0.3 μmol/L), the intervention of YQFM (125, 625, 3,125 μg/ml) reduced cytotoxicity, increased cell viability, inhibited the activity of LDH, elevated adenosine triphosphate (ATP) content and restored mitochondrial membrane potential, which played an anti-apoptotic effect (Zeng et al., 2018). Furthermore, the application of YQFM(2.5 mg/ml) on H9c2 cells significantly boosted cell viability and ATP content in apoptotic cells induced by tert-butyl hydroperoxide, and enhanced the phosphorylation of Akt. It also ameliorated the extent of hypertrophy in H9c2 cells induced by angiotensin II (0.1 μM) and elevated the expression of atrial natriuretic peptide (ANP) mRNA (Zhao C. et al., 2018).

#### 1.2.5 Suppressing inflammatory mediators

In chronic heart failure models induced by the ligation of rats’ left anterior descending coronary artery, after the treatment of YQFM (100 mg/kg/d) for 8 weeks, UPLC-Q-TOF-MS combined with nuclear factor kappa-B (NF-κB) active luciferase reporter analyzed potential anti-inflammatory components. It was further demonstrated that YQFM reduced the size of myocardial infarction, improved cardiac function, and inhibited the expression of inflammatory cytokines, such as tumor necrosis factor-alpha (TNF-α), NF-κB, IL-6, and interleukin-1β (IL-1β). And eight potential anti-inflammatory components have been confirmed, including ginsenosides Rb1, Rg1, Rf, Rh1, Rc, Rb2, Ro and Rg3 (Xing et al., 2013).

#### 1.2.6 Anti-hypoxia effect

To investigate the anti-hypoxia effect of the extraction of YQFM, an animal model of chronic intermittent hypoxia was constructed, treated with YQFM (1.4, 2.8, and 5.5 g/kg/d) for 28 days, and betaloc (0.1516 g/kg/d) served as the positive control. The results manifested that YQFM reversed endothelial cell swelling and cardiac vacuolation, improved myocardial hypoxia tolerance and attenuated myocardial damage by increasing EF and stroke volume (SV), inhibiting the activity of CK and LDH, reducing MDA content, and boosting superoxide dismutase (SOD) (Feng et al., 2016).

#### 1.2.7 Anti-oxidative effect

In ICR mice that were given intraperitoneal injection with isoproterenol (0.02 g/kg/d) for 3 days followed by YQFM (1.352, 0.676 and 0.338 g/kg/d), the results showed that the serum levels of MDA, CK, and LDH and the activity of myeloperoxidase (MPO) decreased, while the serum SOD level elevated, indicating that YQFM exerted great cardioprotective effect (Wang et al., 2013).

#### 1.2.8 Maintaining the balance of matrix metalloproteinases/tissue inhibitor of metalloproteinases

In Wistar rats with chronic heart failure that underwent abdominal aorta contraction, after the intervention of QYFM (520 mg/kg, 260 mg/kg, 1,040 mg/kg) for 14 days, there were significant changes of contents in myocardium, including MMP-2, MMP-3, MMP-9, TIMP-1, TIMP-2. The study reported that YQFM decreased the levels of MMP-2, MMP-3, and MMP-9, and elevated the levels of TIMP-1 and TIMP-2, whereby improving cardiac function and delaying ventricular remodeling (Zhang et al., 2016).

#### 1.2.9 Regulating the expression of miRNAs

In chronic heart failure models established by the ligation of rats’ the left anterior descending coronary artery, after the administration of YQFM (YQFM, 5 mg/kg/d, ip) for 28 days, the differential expression of microRNAs was studied *via* rat miRNA microarray and bioinformatics analysis. And the results manifested that YQFM increased left ventricular ejection fraction (LVEF) and left ventricular fractional shortening (LVFS), decreased left ventricular diameter, and boosted cardiac output (CO) by down-regulating the expression of miR-219a-2-3p, miR-466c-5p, and miR-702-5p, and up-regulating the expression of miR-21-3p, miR-216b-5p, miR-381-3p, and miR-542-3p (Zhao Y. et al., 2018).

Taken together, YQFM reveals great efficacy on delaying myocardial remodeling and improving cardiac function in injured myocardium induced by coronary artery ligation, abdominal aortic constriction, chronic intermittent hypoxia, and doxorubicin. The underlying mechanisms lie in the regulation of mitochondrial function, anti-apoptotic effect on cardiomyocytes, anti-oxidative effect, anti-inflammation, the modulation of the expression of miRNAs, maintenance of MMP/TIMP balance, and anti-hypoxic effect (Table 2).

**TABLE 2:** Myocardial protective effects and mechanisms of YiQiFuMai injection.

Models	Animal/cell	Effects	Mechanisms	Ref
CAL	ICR mice	Improving cardiac function	LDH, CK, MDA, PIIINP, NT-proBNP, HYP↓	Pang et al. (2017)
			Inhibiting the MAPKs signaling pathways	
CAL	ICR mice	Improving mitochondrial function	p-Drp1, NOX2, p67^phox^, NOX4, CACNA1C, p-CaMKII, ROS↓	Zhang et al. (2019)
			Mfn2↑	
CAL	ICR mice	Ameliorating myocardial apoptosis	p-p38, p-JNK, p-ERK1/2↓	Li F. et al. (2019)
			PI3K, p-Akt, p-AMPK↑	
CAL	SD rat	Suppressing inflammatory mediators	TNF-α, NF-κB, IL-6, IL-1β↓	Xing et al. (2013)
CAL	Wistar rat	Regulating the expression of miRNAs	miR-219a-2-3p, miR-466c-5p, miR-702-5p↓	Zhao Y. et al. (2018)
			miR-21-3p, miR-216b-5p, miR-381-3p, miR-542-3p↑	
Abdominal aortic banding	Wistar rat	Improving cardiac function	LVIDs, BNP, CCP↓	Zhang et al. (2015)
			LVPWs, LVEF, LVFS↑	
Abdominal aortic banding	Wistar rat	Improving ventricular remodeling	MMP-2, MMP-3, MMP-9↓	Zhang et al. (2016)
			TIMP-1, TIMP-2↑	
Chronic intermittent hypoxia	ICR mice	Anti-hypoxia	CK, LDH, MDA↓	Feng et al. (2016)
			EF, SV, SOD↑	
Adriamycin	SD rat	Reducing myocardial injury	CK, LDH, AST, LVEDD↓	Wang X. D. et al. (2020)
			FS↑	
Isoprenaline	ICR mice	Anti-oxidative	CK, LDH, MDA, MPO↓	Wang et al. (2013)
			SOD↑	
H_2_O_2_	H9c2 cells	Protecting myocardial cells	Cell survival rate↑	Li et al. (2020)
Adriamycin	H9c2 cells	Anti-apoptosis	LDH, Caspase-3↓	Zeng et al. (2018)
			Cell survival rate, ATP↑	
Angiotensin II	H9c2 cells	Anti-hypertrophy	Surface area of cells, ANP↓	Zhao Y. et al. (2018)
Tert-butyl hydroperoxide	H9c2 cells	Anti-apoptosis	Cell survival rate, ATP, p-Akt↑	Zhao Y. et al. (2018)

Note: ANP, atrialnatriureticpeptide; AST, spartate aminotransferase; ATP, adenosinetriphosphate; BNP, brain natriuretic peptide; CACNA1C, calcium voltage-gated channel subunit α1C; CAL, coronary artery ligation; CK, creatine kinase; CPP, copeptin; EF, ejection fractions; FS, fractional shortening; HYP, hydroxyproline; ICR, institute of cancer research; IL-1β, interleukin-1β; IL-6, interleukin-6; LDH, lactate dehydrogenase; LVEDD, left ventricular end-diastolic diameter; LVEF, left ventricular ejection fraction; LVFS, left ventricular fractional shortening; LVIDs, left ventricular internal diameter in end systole; LVPWs, left ventricular posterior wall in the systolic; MAPKs, mitogen-activated protein kinases; MDA, malondialdehyde; Mfn2, Mitofusin-2; MMP, matrix metalloproteinases; MPO, myeloperoxidase; NADPH, nicotinamide adenine dinucleotide phosphate; NF-κB, nuclear factor kappa-B; NOX2, NADPH, oxidase 2; NOX4, NADPH, oxidase4; NT-proBNP, N-terminal pro-B-type natriuretic peptide; p-Akt, p-protein kinase B; p-AMPK, p-adenine monophosphate activated protein kinase; p-CaMKII, phosphorylation of calmodulin dependent protein kinase II; p-Drp1, phosphorylation of dynamin-related protein 1; p-ERK1/2, p-extracellular signal-regulated kinase 1/2; PI3K, phosphatidylinositol 3-kinase; PIIINP, amino-terminal pro-peptide of pro-collagen type III; p-JNK, p-C-Jun Kinase enzyme; ROS, reactive oxygen species; SOD, superoxide dismutase; SV, stroke volume; TIMP, tissue inhibitor of metalloproteinases; TNF-α, tumor necrosis factor-alpha.

### 1.3 Clinical evidences of YiQiFuMai injection in the treatment of heart failure

#### 1.3.1 Clinical trials

A clinical study involving 1,134 patients with coronary heart disease and heart failure performed by 35 research centers, who were treated with YQFM (5.2 g/d) for 14 days, revealed that the application of YQFM on routine treatment for heart failure contributed to the reduction of Lee’s heart failure score, Minnesota heart failure quality of life score, and cardiothoracic ratio, elevated SV, CO, EF, and FS, and decreased LVESD. Therefore, YQFM exerted great efficacy on improving the cardiac pumping performance and quality of life of patients and reversing ventricular remodeling (Sun et al., 2012). In addition, research has evaluated the clinical efficacy of YQFM combined with western medicine *via* stress echocardiography. A study involving 52 patients with ischemic heart failure showed that, compared with the conventional treatment group, YQFM combined with conventional treatment increased EF and early diastolic peak flow velocity/late diastolic peak flow velocity (E/A) value and reduced early mitral filling velocity/early diastolic mitral annular velocity (E/e') ratio and NT-proBNP levels, indicating that YQFM could improve cardiac function (Hu et al., 2014). Two randomized controlled trials concerning 60 elderly patients with chronic heart failure have illustrated that YQFM combined with basic treatment effectively reduced serum NT-proBNP levels and increased EF and 6-min walking distance (6 MWD) compared to basic treatment alone, suggesting the positive effects of YQFM on cardiac function and exercise tolerance (Zhang et al., 2015; Yang et al., 2016). In another randomized controlled trial involving 108 patients with ischemic cardiomyopathy and heart failure, the treatment group was given YQFM combined with Qiliqiangxin Capsules, while the control group was given Qiliqiangxin Capsules alone. And the results manifested that the application of YQFM lowered serum NT-proBNP levels and elevated EF, CO, and 6 MWD, indicating that YQFM combined with Qiliqiangxin Capsules enhanced clinical efficacy, improved clinical symptoms, and promoted the recovery of cardiac function (Jiang et al., 2018). In a study including 103 patients with chronic heart failure and atrial fibrillation, based on the conventional treatment, the control group was given rosuvastatin while the treatment group was given rosuvastatin and YQFM. Compared to the control group, there were significant improvement of cardiac function and 6 MWD performance, increased FS and EF, and reduction in the level of NT-proBNP, LVEDD, the recurrence rate of atrial fibrillation, and risk of permanent atrial fibrillation in the treatment group (Su and Wang, 2018). In a randomized controlled trial of 118 patients with coronary heart disease and chronic heart failure, the application of YQFM combined with atorvastatin improved cardiac function and delayed ventricular remodeling by decreasing LVEDD, lowering the levels of NT-proBNP, soluble CD40 (sCD40) and soluble CD146 (sCD146), and increasing nitric oxide (NO) levels (Li Y. et al., 2019). Furthermore, in a randomized controlled study involved 40 patients who underwent cardiac valve replacement surgery, based on the cardiac rehabilitation, the treatment group received YQFM immediately after surgery. And the results showed that YQFM effectively improved exercise tolerance and 6 MWD performance (Zhang et al., 2020).

Collectively, YQFM exerts great efficacy in the treatment of heart failure, including improving cardiac function, inhibiting ventricular remodeling, and elevating the quality of life. However, the quality of clinical trials of YQFM in the treatment of heart failure remains relatively low. Thus large-sample, multi-center, randomized controlled trials and real world research are vital to provide the evidence-based data (Table 3).

**TABLE 3:** Clinical trials of YiQiFuMai injection in the treatment of heart failure.

Studies	Control	Treatment	Doses	Periods	Results
Self Controlled Trial					
Sun et al. (2012)		n = 1,134	5.2 g	14 d	LVESD, cardiothoracic ratio, Lee’s heart failure score, Minnesota heart failure score↓
					SV, CO, LVEF, LVFS↑
Hu et al. (2014)		n = 52	5.2 g	14 d	LVEF, E/A↑
					NT-proBNP, E/e’↓
Randomized Controlled Trial					
Zhang P. (2015)	n = 30	n = 30	5.2 g	14 d	NT-proBNP↓
					LVEF, 6 MWD↑
Yang et al. (2016)	n = 30	n = 30	5.2 g	14 d	NT-proBNP↓
					LVEF↑
Jiang et al. (2018)	n = 54	n = 54	5.2 g	14 d	NT-proBNP↓
					LVEF, CO, 6 MWD↑
Su and Wang, (2018)	n = 51	n = 52	2.6–3.9 g	14 d	LVFS, LVEF, 6 MWD↑
					NT-proBNP, LVEDD↓
Li Y. et al. (2019)	n = 59	n = 59	5.2 g	14 d	LVEDD, NT-proBNP, sCD40, sCD146, PAPP-A↓
					NO↑
Zhang et al. (2020)	n = 20	n = 20	5.2 g	(9.05 ± 2.74)d	6 MWD↑

Note: CO, cardiac output; E/A, early diastolic peak flow velocity/late diastolic peak flow velocity; E/e', early mitral filling velocity/early diastolic mitral annular velocity; LVEDD, left ventricular end diastolic inner diameter; LVESD, left ventricular end systolic diameter; LVEF, left ventricular ejection fraction; LVFS, left ventricular fractional shortening; NT-proBNP, N-terminal pro-B-type natriuretic peptide; NO, nitric oxide; PAPP-A, pregnancy associated plasma protein-A; sCD146, soluble CD146; sCD40, soluble CD40; SV, stroke volume; 6 MWD, 6-min walking distance.

#### 1.3.2 Meta-analyse and systematic reviews

Meta-analysis evaluating clinical efficacy of YQFM combined with conventional western medicine in the treatment of heart failure has revealed that the intervention of YQFM increased EF, CO and 6 MWD, shortened LVEDD and LVESD, and reduced the serum levels of NT-proBNP and BNP, suggesting that on the basis of conventional western medicine treatment, YQFM further improved cardiac function and the quality of life (Wang XL. et al., 2016; Lian et al., 2016; Zhou et al., 2016; Xiong et al., 2017; Xie and Dai, 2019; Fan et al., 2021). Whereas, lacking of the calculation of sample size, details concerned the western medicine treatment and endpoint indicators, unclear random method, and non-uniform dosages and periods of YQFM weaken the quality of the clinical evidence (Table 4).

**TABLE 4:** Meta-analyse and systematic reviews of YiQiFuMai injection in the treatment of heart failure.

Studies	Populations	Control	Treatment	Outcome indicators	Clinical efficacy
Wang Y. et al. (2016)	Chronic heart failure	893	1,046	Clinical comprehensive efficacy, NYHA classification, LVEF, LVEDD, NT-proBNP	OR = 3.63
					95%*CI* (2.22, 5.93)
Lian et al. (2016)	Heart failure	493	508	Clinical comprehensive efficacy, LVEF, LVESD, CO, NT-proBNP, 6 MWD	OR = 3.09
					95%*CI* (2.05, 4.67)
Zhou et al. (2016)	Heart failure	433	446	Overall effective rate, LVEF, LVEDD, BNP	OR = 4.45
					95%*CI* (2.62, 7.56)
Xiong et al. (2017)	Heart failure	1,180	1,201	Overall effective rate, LVEF, LVEDD, CO, BNP, 6 MWD	OR = 2.96
					95%*CI* (2.23, 3.94)
Xie and Dai, (2019)	Heart failure	961	976	Clinical comprehensive efficacy, LVEF, LVEDD, 6 MWD, NT-pro BNP, BNP	RR = 3.06
					95%*CI* (2.27, 4.12)
Fan et al. (2021)	Heart failure	1,475	1,502	Clinical comprehensive efficacy, LVEF, BNP, NT-proBNP, E/A ratio, CO, LVEDD, 6 MWT	RR = 1.21
					95%*CI* (1.16, 1.25)

Note: BNP, B-type natriuretic peptide; CO, cardiac output; LVEDD, left ventricular end diastolic inner diameter; LVEF, left ventricular ejection fraction; LVESD, left ventricular end systolic diameter; NT-pro BNP, N-terminal pro-B-type natriuretic peptide; 6 MWD, 6-min walking distance.

### 1.4 Safety of YiQiFuMai injection in the treatment of heart failure

Li *et al.* analyzed 240 patients with coronary heart disease who received YQFM in retrospect, and the results showed that none of the patients had the symptoms or signs of hepatic and renal injury. It was merely reported one case of pharyngeal pain with the injection of YQFM infusion, and the symptom subsided gradually after the withdrawn of YQFM (Li et al., 2018). An analysis involving 998 patients treated with YQFM (>70 years old accounting for 50.69%) exhibited that the incidence of untoward effects was 0.2%, and most of these symptoms and signs were transient without additional treatment (Li K. et al., 2019). Wang *et al.* analyzed the safety of YQFM on 106 elderly patients (≥80 years old) with cardiovascular diseases, and it was found that YQFM had little impact on the levels of serum alanine aminotransferase (ALT), AST, total bilirubin (TBil), and creatinine (Cr), and only one case (accounting for 0.94%) reported mild palpitation and precordial discomfort (Wang et al., 2018). Sun *et al.* retrospectively analyzed the performance of YQFM on 2,476 hospitalized patients by the prescription automatic screening system. The study reported that 31 cases had adverse reactions (accounting for 1.25%) that mainly manifested as general damage and skin lesion, such as rash, pruritus, chills, high fever, *etc.* Additionally, most adverse reactions occurred within 7 days after the medication (Sun et al., 2019). Cao *et al.* conducted a prospective, large-sample, multi-center analysis involving 44 medical institutions, including 10,767 hospitalized patients treated with YQFM. The research reported 19 cases of adverse reactions (0.176%), and among them, skin lesion was the main one (36.98%). At the same time, it seemed that adverse reactions were more common in the group with higher infusion rate (*p* < 0.05), indicating that immoderate infusion rate was one of the risk factors for untoward effects (Cao H. B. et al., 2019). Ma *et al.* analyzed 51 pieces of clinical research involving 7,824 patients who were given YQFM. There were no serious adverse event, and only 23 study mentioned 42 cases of mildly untoward effects in total. Among them, adverse cardiovascular events, adverse neural damage, skin lesion, and gastrointestinal reactions accounted for 28.6%, 20.4%, 22.5%, and 20.3% respectively (Ma et al., 2015). *In vitro* experiments, YQFM not only inhibited the autonomic contraction of the isolated intestine, but also supressed the spasm of isolated intestine triggered by acetylcholine (Ach) and histamine (His). *In vivo* experiments the blue staining rate and the levels of His and 5-hydroxytryptamine (5-HT) in mice administered with low-dose YQFM were within the normal range without evident pulmonary injury and auricularinfection. By contrast, only when the mice were given 3.43 times the clinical equivalent dose of YQFM, there were mild increase in the blue staining rate and the levels of His and 5-hydroxytryptamine (5-HT) and inflammation, indicating that YQFM had few allergic reactions within a proper dosage range (Gu et al., 2018). Clinical studies have also verified that adverse reactions of YQFM mainly correlated with inappropriate prescription, including beyond indications and dosages, unnecessary treatment, contraindications, and excessive quantities of solvent, etc (Zhao C. et al., 2018). Though YQFM reveals great safety and efficacy, it is worth noting that following the instructions strictly is the key to avoid adverse events.

## 2 Conclusion

**FIGURE 1:**
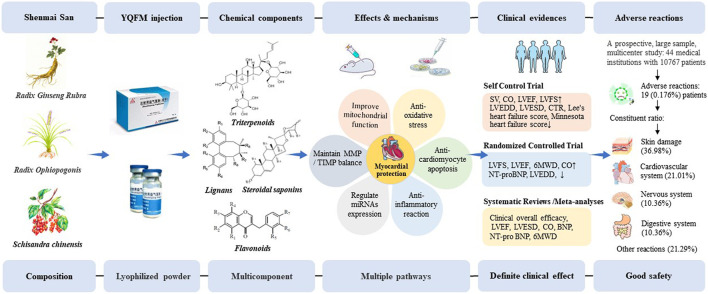
The process of YQFM in treating heart failure. BNP, brain natriuretic peptide; CO, cardiac output; CTR, calcitonin receptor; LVEDD, left ventricular end-diastolic diameter; LVEF, left ventricular ejection fraction; LVESD, left ventricular end-systolic diameter; LVFS, left ventricular fractional shortening; MMP/TIMP, matrix metalloproteinases/tissue inhibitor of metalloproteinases; NT-proBNP, N-terminal pro-B-type natriuretic peptide; SV, stroke volume; 6MWD, 6-min walking distance.

Heart failure, the cumulative effect and endpoint of various cardiac abnormalities, eventually leads to the decline of cardiac pump function, putting up with the challenge to the exploration of effective strategy for the prevention and treatment of heart failure (Committee of Exports on Rational Drug Use National Health and Family Planning Commission of The People’Republic of China and Chinese Pharmacists Association, 2019). Long-term clinical practice verified that Traditional Chinese medicine exerts the composite effect through the multi-target and multi-link ways (Zhang RP., 2015). At present, there are plenty of fundamental research and clinical trials on YQFM, involving pharmacodynamic components, pharmacological effects, clinical application and quality markers. Research has demonstrated that YQFM improved cardiac function, inhibited ventricular remodeling, exerted great anti-inflammatory and anti-oxidative effect, regulated mitochondrial function, thereby improving the quality of life of patients with heart failure (Du et al., 2021). YQFM is widely used in the treatment of heart failure, with definite clinical efficacy and fewer adverse reactions, which provides a reference for rational clinical drug use (Figure 1).

However, there are great gaps referring to the dose-effect relationship, pharmacological targets and mechanism of YQFM in the treatment of cardiovascular diseases waiting to be filled in. The multi-component and multi-target characteristics of Traditional Chinese Medicine raise the bar for the exploration of pharmacological mechanism of YQFM in the treatment of heart failure. At present, the pharmacological effects of different components of YQFM on heart failure remains unclear, and the research merely focuses on the study of ginsenosides. However, there are few studies on the pharmacological effects, mechanisms and targets of the two traditional Chinese medicines of Radix of *Ophiopogon japonicus* (Thunb.) Ker Gawl (Liliaceae), and Fructus of *Schisandra chinensis* (Turcz.) Baill (Schisandraceae), as well as the important active components of Ophiopogonins, Ophiopogon japonicus polysaccharide and Schizandrin A. What’s more, in some studies the dosage of YQFM is not adaptive for the clinical treatment, resulting in the mismatch between clinical practice and basic science. In the future, research ought to reveal the targets and related signaling pathways of YQFM and differ active components so as to provide scientific guidance for the application of YQFM in clinical practice. Besides, owning to the relatively low quality of clinical trials on YQFM in the treatment of heart failure, large-scale, multi-center, high-quality randomized controlled clinical trials and real world studies are badly in need. Finally, although there are few reports about the adverse effects of YQFM, non-standard prescription still an annoying epidemic in the clinical practice. Therefore, it is necessary to improve the legal system of drug reevaluation and the post-marketing supervision to guard the safety and efficacy of the application of drugs, so as to improve the efficacy of YQFM in the treatment of heart failure.

## Glossary

Ach: acetylcholine
Akt: protein kinase B
ALT: alanine aminotransferase
AMPK: adenine monophosphate activated protein kinase
ANP: atrialnatriureticpeptide
ARNI: angiotensin receptor neprilysin inhibitor
AST: aspartate aminotransferase
ATP: adenosinetriphosphate
BNP: brain natriuretic peptide
CACNA1C: calcium voltage-gated channel subunit α1C
CAL: coronary artery ligation
CK: creatine kinase
CK-MB: creatine kinase-MB
CO: cardiac output
CPP: copeptin
Cr: creatinine
Drp1: dynamin-related protein one
E/A: early diastolic peak flow velocity/late diastolic peak flow velocity
E/e': early mitral filling velocity/early diastolic mitral annular velocity
EF: ejection fraction
ERK1/2: extracellular signal-regulated kinase 1/2
FS: fractional shortening
His: histamine
HPLC: high-performance liquid chromatography
HPLC-Q-TOF-MS: HPLC coupled with quadrupole time-of-flight tandem mass spectrometry
HYP: hydroxyproline
IL-1β: interleukin-1β
IL-6: interleukin-6
JNK: C-Jun Kinase enzyme
LC-ESI-MS: liquid chromatography electrospray ionization mass spectrometry
LDH: lactic dehydrogenase
LMS: liquid-mass spectrometry
LVEDD: left ventricular end-diastolic diameter
LVEF: left ventricular ejection fraction
LVESD: left ventricular end-systolic diameter
LVPWs: left ventricular posterior wall in the systolic
LVFS: left ventricular fractional shortening
MAPKs: mitogen-activated protein kinases
MDA: malondialdehyde
Mfn2: mitochondrial fusion protein two
MMP/TIMP: matrix metalloproteinases/tissue inhibitor of metalloproteinases
MPO: myeloperoxidase
NF-κB: nuclear factor kappa-B
NO: nitric oxide
NOX2: NADPH oxidase two
NT-proBNP: N-terminal pro-B-type natriuretic peptide
p-CaMKII: phosphorylation of calmodulin dependent protein kinase II
PI3K: phosphatidylinositol 3-kinase
PIIINP: amino-terminal pro-peptide of pro-collagen type III
ROS: reactive oxygen species
sCD146: soluble CD146
sCD40: soluble CD40
SGLT2i: sodium glucose cotransporter two inhibitor
SOD: superoxide dismutase
SV: stroke volume
TBil: total bilirubin
TNF-α: tumor necrosis factor-alpha
UFLC-IT-TOF/MS: ultrafast liquid chromatography-ion trap time-of-flight MS
UPLC-Q-TOF/MS: ultra-performance liquid chromatography coupled with quadrupole time-of-flight tandem mass spectrometry
YQFM: the YiQiFuMai injection
5-HT: 5-hydroxytryptamine
6 MWD: 6-min walking distance.
